# (Nitrato-κ^2^ *O*,*O*′)bis­(1,10-phenanthroline-κ^2^ *N*,*N*′)copper(II) tricyano­methanide

**DOI:** 10.1107/S1600536812047757

**Published:** 2012-11-28

**Authors:** Katarína Lacková, Ivan Potočňák

**Affiliations:** a Department of Inorganic Chemistry, Faculty of Science, P.J. Šafárik University, Moyzesova 11, SK-041 54 Košice, Slovakia

## Abstract

The title compound, [Cu(NO_3_)(C_12_H_8_N_2_)_2_][C(CN)_3_], is formed of discrete [Cu(NO_3_)(phen)_2_]^+^ complex cations (phen is 1,10-phenanthroline) and C(CN)_3_^−^ counter-anions. The Cu^II^ atom has an asymmetric tetragonal–bipyramidal (4 + 1+1) stereochemistry with a pseudo-*C*_2_ symmetry axis bis­ecting the nitrate ligand and passing through the Cu^II^ atom between the two phen ligands. The four N atoms of the phen ligands coordinate to the Cu^II^ atom with Cu—N distances in the range 1.974 (2)–2.126 (2) Å, while the two O atoms coordinate at substanti­ally different distances [2.154 (2) and 2.586 (2) Å]. The structure is stabilized by C—H⋯O hydrogen bonds and weak π–π inter­actions between nearly parallel benzene and pyridine rings of two adjacent phen mol­ecules, with centroid–centroid distances of 3.684 (2) and 3.6111 (2) Å, and between π-electrons of the tricyano­methanide anion and the pyridine or benzene rings [N⋯(ring centroid) distances = 3.553 (3)–3.875 (3) Å].

## Related literature

For five-coordinate Cu^II^ in [Cu(*L*)_2_ *X*]*Y* complexes [*L* = 1,10-phenanthroline (phen) or 2,2′-bipyridine (bpy); *X* = N(CN)_2_^−^ or ONC(CN)_2_^−^, *Y* = 1^−^ anion], see: Potočňák *et al.* (2005 ▶, 2008 ▶). For complexes containing [Cu(NO_3_)(phen)_2_]^+^ cations, see: van Meerssche *et al.* (1981 ▶); Marsh (1997 ▶); Chen *et al.* (2005 ▶); Ovens *et al.* (2010 ▶). For complexes containing [Cu(bpy)_2_NO_3_]^+^ cations, see: Prasad *et al.* (1999 ▶); Marjani *et al.* (2005 ▶). For π–π inter­actions, see: Janiak (2000 ▶). For a description of the properties of the tricyano­methanide (tcm or C(CN)_3_^−^) anion, see: Golub *et al.* (1986 ▶). For [Cu(*L*)_2_ *Y*]tcm (*Y* = Cl^−^ or Br^−^), see: Lacková (2012 ▶).
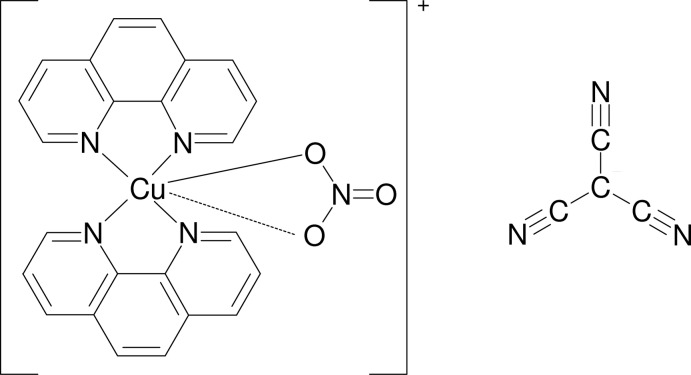


## Experimental

### 

#### Crystal data

[Cu(NO_3_)(C_12_H_8_N_2_)_2_](C_4_N_3_)
*M* *_r_* = 576.03
Monoclinic, 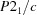

*a* = 13.3011 (4) Å
*b* = 10.1155 (3) Å
*c* = 19.5597 (6) Å
β = 103.997 (3)°
*V* = 2553.56 (13) Å^3^
*Z* = 4
Mo *K*α radiation
μ = 0.90 mm^−1^
*T* = 183 K
0.38 × 0.31 × 0.26 mm

#### Data collection

Oxford Diffraction Xcalibur Sapphire2 diffractometer
Absorption correction: analytical [*CrysAlis RED* (Oxford Diffraction, 2007 ▶), based on expressions derived from Clark & Reid (1995 ▶)] *T*_min_ = 0.777, *T*_max_ = 0.825
10675 measured reflections
5283 independent reflections
4034 reflections with *I* > 2σ(*I*)
*R*_int_ = 0.024

#### Refinement

*R*[*F* ^2^ > 2σ(*F* ^2^)] = 0.040
*wR*(*F* ^2^) = 0.097
*S* = 1.01
5283 reflections
361 parameters
H-atom parameters constrained
Δρ_max_ = 0.40 e Å^−3^
Δρ_min_ = −0.43 e Å^−3^

### 

Data collection: *CrysAlis CCD* (Oxford Diffraction, 2007 ▶); cell refinement: *CrysAlis RED* (Oxford Diffraction, 2007 ▶); data reduction: *CrysAlis RED*; program(s) used to solve structure: *SHELXS97* (Sheldrick, 2008 ▶); program(s) used to refine structure: *SHELXL97* (Sheldrick, 2008 ▶); molecular graphics: *DIAMOND* (Brandenburg, 2001 ▶); software used to prepare material for publication: *SHELXL97*.

## Supplementary Material

Crystal structure: contains datablock(s) I, global. DOI: 10.1107/S1600536812047757/gk2531sup1.cif

Structure factors: contains datablock(s) I. DOI: 10.1107/S1600536812047757/gk2531Isup2.hkl

Additional supplementary materials:  crystallographic information; 3D view; checkCIF report

## Figures and Tables

**Table 1:** Hydrogen-bond geometry (Å, °)

*D*—H⋯*A*	*D*—H	H⋯*A*	*D*⋯*A*	*D*—H⋯*A*
C12—H12⋯O3^i^	0.93	2.52	3.206 (3)	131
C24—H24⋯O2^ii^	0.93	2.44	3.231 (3)	144
C36—H36⋯O3^iii^	0.93	2.45	3.307 (3)	153
C46—H46⋯O1^iv^	0.93	2.47	3.201 (3)	136

Symmetry codes: (i) 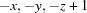; (ii) 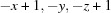; (iii) 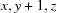; (iv) 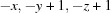.

**Fig. 1.**
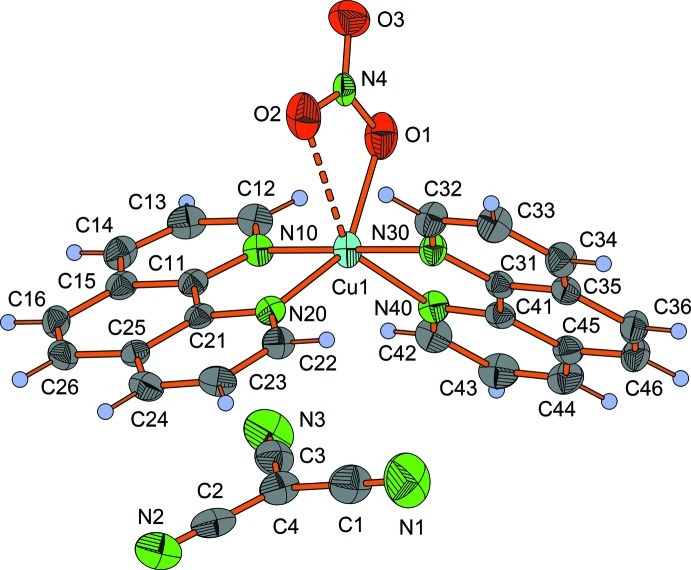
The structure of the title compound. Displacement ellipsoids are drawn at the 50 % probability for non-H atoms. H atoms are represented as small spheres of arbitrary radius.

**Fig. 2.**
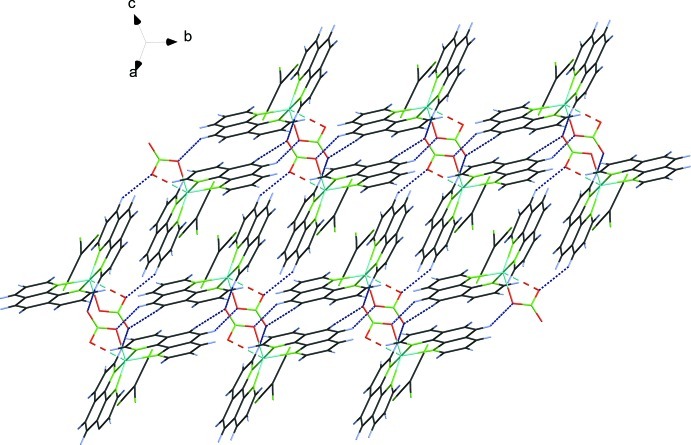
C–H···O hydrogen bonds (dashed lines) in the title compound.

**Fig. 3.**
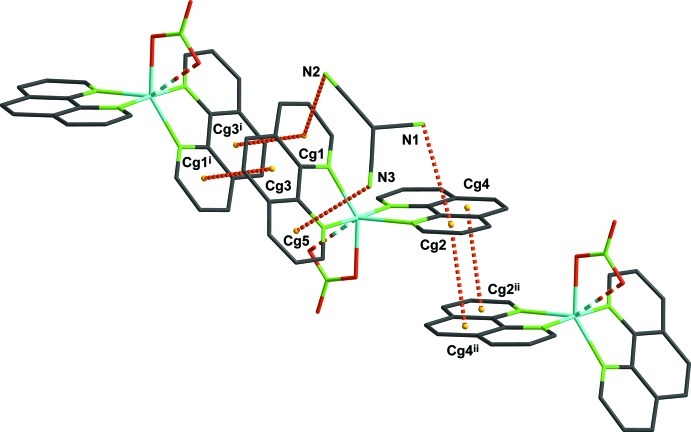
π-π interactions (dashed lines) between tcm, benzene and pyridine rings in the title compound (symmetry codes: (i) = 1 - x, -y, 1 - z; (ii) = -x, 1 - y, 1 - z).

## supplementary crystallographic information

### Comment

The shape of coordination polyhedra (SCP) in the case of five-coordination is one of the current problems in coordination chemistry. With the aim of establishing possible reasons for different SCP in related compounds, our research group has previously prepared and studied the structures of five-coordinate copper(II) complexes of the general formula [Cu(*L*)_2_*X*]*Y*, where *L* is 1,10-phenanthroline (phen) or 2,2'-bipyridine (bpy), *X* is N(CN)_2_^-^ or ONC(CN)_2_^-^ and *Y* is 1- anion (Potočňák *et al.*, 2005, 2008). The obtained results showed that the preferred SCP for compounds with phen is close to trigonal bipyramid, whereas SCP for bpy compounds is close to tetragonal pyramid. It is known that tricyanomethanide anion (tcm, C(CN)_3_^-^) can coordinate similarly as N(CN)_2_^-^ and ONC(CN)_2_^-^ anions (Golub *et al.*, 1986). Thus, to verify the findings about SCP, we have attempted to prepare compounds with *X* = tcm and *Y* = Cl^-^ or Br^-^ and checked their SCP. However, X-ray structure analysis of four prepared complexes has shown that their formulae are [Cu(*L*)_2_*Y*]tcm, thus smaller Cl^-^ and Br^-^ anions were coordinated while larger tcm anion remained out of coordination sphere (Lacková, 2012). Therefore, we have decided to replace smaller Cl^-^ or Br^-^ anions by larger NO_3_^-^ anion with the hope that it remains uncoordinated. Nevertheless, the title compound, [Cu(NO_3_)(phen)_2_]tcm with coordinated NO_3_^-^ and uncoordinated tcm anions has been prepared during our attempts and we present its structure here.

The title compound is formed by discrete [Cu(NO_3_)(phen)_2_]^+^ cation and an uncoordinated C(CN)_3_^-^ anion (Figure 1). The Cu^II^ atom is coordinated by four nitrogen atoms from two phen ligands at average Cu–N distance of 2.03 (7) Å and O1 atom from the nitrato ligand at 2.154 (2) Å. The second nitrato oxygen atom (O2) is 2.586 (2) Å away from the Cu^II^ atom, thus it can be considered as semi-coordinated; similar values have been observed in the [Cu(bpy)_2_NO_3_]NO_3_ (2.138 (6) and 2.520 (6) Å) (Marjani *et al.*, 2005) and [Cu(bpy)_2_(NO_3_)]NO_3_^.^HDCI^.^H_2_O, HDCI = 4,5-dicyanoimidazole (2.078 (3) and 2.639 (4) Å) (Prasad *et al.*, 1999) compounds. Thus, the Cu^II^ atom in the title compound has an asymmetric tetragonal–bipyramidal (4+1+1) stereochemistry with pseudo-*C*_2_ symmetry axis bisecting the NO_3_ ligand and passing between the two phen ligands. On the other hand, the two Cu–O distances in known [Cu(NO_3_)(phen)_2_]*Y* complexes are much closer to each other [2.1549 (5) and 2.4886 (5) Å for *Y* = CCl_3_COO^-^ (van Meerssche *et al.*, 1981), 2.3119 (5) and 2.3119 (5) Å for *Y* = CCl_3_COO^-^ (Marsh, 1997), 2.0137 (3) and 2.3316 (3) Å for *Y* = OH^-^ (Chen *et al.*, 2005) and 2.302 (6) and 2.416 (6) Å for *Y* = [AuBr_2_(CN)_2_]^-^ (Ovens *et al.*, 2010)].

Each of the two phen molecules in the title compound possesses one nitrogen atom (N20 and N40) occupying an equatorial position and one nitrogen atom (N10 and N30) coordinated in an axial position (corresponding bond lengths are reported in Table of geometric parameters), two remaining equatorial positions are occupied by O1 and O2 atoms from the nitrato ligand. Aromatic rings of phen molecules are nearly planar; the largest deviation of atoms from their mean planes is 0.060 (3) Å for C13 atom and bond distances and angles are normal (van Meerssche *et al.*, 1981; Marsh, 1997; Chen *et al.*, 2005; Ovens *et al.*, 2010). The positive charge of the [Cu(NO_3_)(phen)_2_]^+^ cation is balanced by an uncoordinated tcm anion, which is settled under the "umbrella" of the copper atom and the two phen molecules. The N≡C as well as the C–C distances (Table of geometric parameters) are usual for triple N≡C (1.15 Å) and single C–C (1.40 Å) bonds (Golub *et al.*, 1986). The bond angles around methanide and cyanide carbon atoms are, as expected, nearly 120 and 180° confirming *sp*^2^ and *sp* hybridization states of the corresponding carbon atoms (Table of geometric parameters).

The structure of the title compound is stabilized by weak C–H···O hydrogen bonds (Table 1, Figure 2) which link the cations and anions into the plane parallel with (001). The next stabilization comes from two kinds of π-π interactions (Janiak, 2000). There are face to face π-π interactions between nearly planar benzene and pyridine rings [*Cg*1^i^ (N20^i^, C21^i^ – C25^i^)···*Cg*3 (C11, C15, C16, C26, C25, C21) = 3.684 (2) Å; *Cg*2 (N40, C41 – C45)···*Cg*4^ii^ (C31^ii^, C35^ii^, C36^ii^, C46^ii^, C45^ii^, C41^ii^) = 3.611 (2) Å (*Cg* = centroid); symmetry codes: (i) = 1 - *x*, -*y*, 1 - *z*; (ii) = -*x*, 1 - *y*, 1 - *z*] and π-π interaction between π electrons of the tcm anion and the benzene or pyridine rings [N1···*Cg*2 = 3.654 (4) Å; N2···*Cg*1 (N20, C21 – C25) = 3.875 (3) Å; N3···*Cg*5 (N10, C11 – C15)= 3.553 (3) Å] as shown in Figure 3.

### Experimental

The title compound was prepared by chance during our attempts to prepare [Cu(phen)_2_(tcm)]NO_3_ compound with a five-coordinated Cu^II^ atom. Crystals of the title compound were prepared by mixing a 0.1 *M* aqueous solution of Cu(NO_3_)_2_ (5 ml, 0.5 mmol) with a 0.1 *M* methanol solution of 1,10-phenanthroline (10 ml, 1 mmol). To the resulting green solution, a 0.1 *M* aqueous solution of KC(CN)_3_ (5 ml, 0.5 mmol) was added (all solutions were heated almost to boiling before mixing). After 15 days, green crystals were filtered off and dried in air.

### Refinement

H-atoms were positioned geometrically and refined as riding atoms, with C–H = 0.93 Å and *U*_iso_(H) = 1.2*U*_eq_(C).

### Crystal data

**Table:**

[Cu(NO_3_)(C_12_H_8_N_2_)_2_](C_4_N_3_)	*F*(000) = 1172
*M**_r_* = 576.03	*D*_x_ = 1.498 Mg m^−^^3^
Monoclinic, *P*2_1_/*c*	Mo *K*α radiation, λ = 0.71069 Å
Hall symbol: -P 2ybc	Cell parameters from 4424 reflections
*a* = 13.3011 (4) Å	θ = 2.9–29.4°
*b* = 10.1155 (3) Å	µ = 0.90 mm^−^^1^
*c* = 19.5597 (6) Å	*T* = 183 K
β = 103.997 (3)°	Prism, green
*V* = 2553.56 (13) Å^3^	0.38 × 0.31 × 0.26 mm
*Z* = 4	

### Data collection

**Table:**

Oxford Diffraction Xcalibur Sapphire2 diffractometer	5283 independent reflections
Radiation source: fine-focus sealed tube	4034 reflections with *I* > 2σ(*I*)
Graphite monochromator	*R*_int_ = 0.024
Detector resolution: 8.3438 pixels mm^-1^	θ_max_ = 26.5°, θ_min_ = 2.9°
ω scans	*h* = −16→9
Absorption correction: analytical [*CrysAlis RED* (Oxford Diffraction, 2007), based on expressions derived from Clark & Reid (1995)]	*k* = −11→12
*T*_min_ = 0.777, *T*_max_ = 0.825	*l* = −22→24
10675 measured reflections	

### Refinement

**Table:**

Refinement on *F*^2^	Primary atom site location: structure-invariant direct methods
Least-squares matrix: full	Secondary atom site location: difference Fourier map
*R*[*F*^2^ > 2σ(*F*^2^)] = 0.040	Hydrogen site location: inferred from neighbouring sites
*wR*(*F*^2^) = 0.097	H-atom parameters constrained
*S* = 1.01	*w* = 1/[σ^2^(*F*_o_^2^) + (0.0384*P*)^2^ + 1.6904*P*] where *P* = (*F*_o_^2^ + 2*F*_c_^2^)/3
5283 reflections	(Δ/σ)_max_ = 0.001
361 parameters	Δρ_max_ = 0.40 e Å^−^^3^
0 restraints	Δρ_min_ = −0.43 e Å^−^^3^

### Special details

**Table:**

Geometry. All e.s.d.'s (except the e.s.d. in the dihedral angle between two l.s. planes) are estimated using the full covariance matrix. The cell e.s.d.'s are taken into account individually in the estimation of e.s.d.'s in distances, angles and torsion angles; correlations between e.s.d.'s in cell parameters are only used when they are defined by crystal symmetry. An approximate (isotropic) treatment of cell e.s.d.'s is used for estimating e.s.d.'s involving l.s. planes.
Refinement. Refinement of *F*^2^ against ALL reflections. The weighted *R*-factor *wR* and goodness of fit *S* are based on *F*^2^, conventional *R*-factors *R* are based on *F*, with *F* set to zero for negative *F*^2^. The threshold expression of *F*^2^ > σ(*F*^2^) is used only for calculating *R*-factors(gt) *etc*. and is not relevant to the choice of reflections for refinement. *R*-factors based on *F*^2^ are statistically about twice as large as those based on *F*, and *R*- factors based on ALL data will be even larger.

### Fractional atomic coordinates and isotropic or equivalent isotropic displacement parameters (Å^2^)

**Table:**

	*x*	*y*	*z*	*U*_iso_*/*U*_eq_	
Cu1	0.23397 (2)	0.23042 (3)	0.492800 (16)	0.02860 (11)	
N10	0.20297 (15)	0.0829 (2)	0.42426 (10)	0.0279 (5)	
N20	0.37950 (15)	0.2104 (2)	0.47543 (10)	0.0254 (4)	
N30	0.27128 (15)	0.3747 (2)	0.56231 (10)	0.0269 (5)	
N40	0.12822 (15)	0.3762 (2)	0.43990 (10)	0.0273 (5)	
O1	0.12472 (15)	0.1517 (2)	0.54749 (10)	0.0456 (5)	
O2	0.27099 (14)	0.0638 (2)	0.59615 (12)	0.0480 (5)	
O3	0.13425 (17)	0.0080 (2)	0.62999 (11)	0.0535 (6)	
N4	0.17688 (15)	0.07347 (19)	0.59182 (11)	0.0251 (5)	
C11	0.28763 (18)	0.0376 (2)	0.40462 (12)	0.0247 (5)	
C12	0.1122 (2)	0.0234 (3)	0.39845 (13)	0.0326 (6)	
H12	0.0538	0.0542	0.4117	0.039*	
C13	0.1022 (2)	−0.0837 (3)	0.35220 (14)	0.0358 (6)	
H13	0.0376	−0.1216	0.3340	0.043*	
C14	0.1878 (2)	−0.1322 (3)	0.33397 (13)	0.0341 (6)	
H14	0.1820	−0.2053	0.3045	0.041*	
C15	0.2850 (2)	−0.0715 (2)	0.35975 (12)	0.0280 (6)	
C16	0.3793 (2)	−0.1106 (3)	0.34200 (13)	0.0316 (6)	
H16	0.3789	−0.1841	0.3134	0.038*	
C21	0.38273 (18)	0.1067 (2)	0.43158 (12)	0.0236 (5)	
C22	0.46619 (19)	0.2782 (3)	0.49966 (13)	0.0302 (6)	
H22	0.4648	0.3495	0.5294	0.036*	
C23	0.5594 (2)	0.2471 (3)	0.48255 (13)	0.0320 (6)	
H23	0.6186	0.2971	0.5007	0.038*	
C24	0.5630 (2)	0.1427 (3)	0.43896 (13)	0.0319 (6)	
H24	0.6248	0.1209	0.4273	0.038*	
C25	0.47279 (19)	0.0682 (2)	0.41169 (12)	0.0251 (5)	
C26	0.4683 (2)	−0.0431 (3)	0.36593 (13)	0.0311 (6)	
H26	0.5277	−0.0694	0.3525	0.037*	
C31	0.21763 (18)	0.4890 (2)	0.54394 (12)	0.0240 (5)	
C32	0.3421 (2)	0.3704 (3)	0.62327 (13)	0.0327 (6)	
H32	0.3780	0.2919	0.6365	0.039*	
C33	0.3646 (2)	0.4792 (3)	0.66803 (14)	0.0350 (6)	
H33	0.4153	0.4735	0.7101	0.042*	
C34	0.3117 (2)	0.5938 (3)	0.64957 (13)	0.0327 (6)	
H34	0.3265	0.6673	0.6789	0.039*	
C35	0.23491 (19)	0.6016 (2)	0.58662 (13)	0.0267 (5)	
C36	0.1738 (2)	0.7169 (3)	0.56400 (14)	0.0322 (6)	
H36	0.1847	0.7924	0.5920	0.039*	
C41	0.14034 (18)	0.4896 (2)	0.47829 (13)	0.0252 (5)	
C42	0.0572 (2)	0.3760 (3)	0.37912 (13)	0.0348 (6)	
H42	0.0484	0.2998	0.3517	0.042*	
C43	−0.0048 (2)	0.4860 (3)	0.35470 (14)	0.0378 (7)	
H43	−0.0538	0.4821	0.3118	0.045*	
C44	0.0065 (2)	0.5983 (3)	0.39360 (14)	0.0351 (6)	
H44	−0.0352	0.6713	0.3778	0.042*	
C45	0.08147 (18)	0.6040 (3)	0.45786 (13)	0.0282 (6)	
C46	0.1005 (2)	0.7181 (3)	0.50269 (15)	0.0340 (6)	
H46	0.0615	0.7943	0.4893	0.041*	
C1	0.3205 (3)	0.4583 (3)	0.34844 (17)	0.0488 (8)	
C2	0.4051 (2)	0.3074 (3)	0.28228 (14)	0.0384 (7)	
C3	0.2207 (2)	0.2859 (3)	0.27295 (14)	0.0408 (7)	
C4	0.3157 (2)	0.3509 (3)	0.30173 (14)	0.0381 (7)	
N1	0.3254 (3)	0.5442 (3)	0.38713 (17)	0.0772 (10)	
N2	0.4774 (2)	0.2697 (3)	0.26576 (13)	0.0481 (7)	
N3	0.1445 (2)	0.2311 (3)	0.25020 (14)	0.0594 (8)	

### Atomic displacement parameters (Å^2^)

**Table:**

	*U*^11^	*U*^22^	*U*^33^	*U*^12^	*U*^13^	*U*^23^
Cu1	0.02404 (17)	0.02999 (18)	0.02883 (17)	0.00306 (14)	0.00066 (12)	−0.00604 (14)
N10	0.0248 (11)	0.0302 (12)	0.0268 (11)	0.0005 (9)	0.0026 (9)	−0.0015 (9)
N20	0.0246 (10)	0.0265 (11)	0.0233 (10)	−0.0003 (9)	0.0026 (9)	0.0008 (9)
N30	0.0253 (11)	0.0267 (11)	0.0268 (11)	0.0050 (9)	0.0027 (9)	−0.0008 (9)
N40	0.0210 (10)	0.0315 (12)	0.0270 (11)	0.0020 (9)	0.0007 (9)	0.0003 (9)
O1	0.0396 (11)	0.0472 (12)	0.0433 (11)	0.0154 (10)	−0.0029 (9)	−0.0071 (10)
O2	0.0230 (10)	0.0497 (13)	0.0711 (14)	0.0028 (9)	0.0111 (10)	−0.0105 (11)
O3	0.0639 (15)	0.0471 (13)	0.0574 (14)	−0.0118 (11)	0.0298 (12)	−0.0044 (11)
N4	0.0220 (11)	0.0207 (11)	0.0337 (11)	−0.0005 (9)	0.0092 (9)	−0.0091 (9)
C11	0.0266 (13)	0.0253 (13)	0.0203 (12)	0.0015 (11)	0.0019 (10)	0.0032 (10)
C12	0.0279 (13)	0.0373 (15)	0.0305 (14)	−0.0031 (12)	0.0032 (11)	−0.0003 (12)
C13	0.0337 (15)	0.0374 (16)	0.0332 (14)	−0.0103 (13)	0.0018 (12)	−0.0005 (12)
C14	0.0465 (17)	0.0266 (14)	0.0267 (13)	−0.0054 (12)	0.0038 (12)	−0.0056 (11)
C15	0.0355 (14)	0.0257 (13)	0.0210 (12)	0.0002 (11)	0.0030 (11)	0.0025 (10)
C16	0.0454 (16)	0.0263 (13)	0.0233 (12)	0.0063 (12)	0.0090 (12)	−0.0021 (11)
C21	0.0279 (13)	0.0232 (12)	0.0181 (11)	0.0019 (10)	0.0024 (10)	0.0055 (10)
C22	0.0309 (14)	0.0302 (14)	0.0281 (13)	−0.0042 (12)	0.0044 (11)	−0.0029 (11)
C23	0.0262 (13)	0.0402 (16)	0.0284 (13)	−0.0071 (12)	0.0040 (11)	0.0023 (12)
C24	0.0263 (13)	0.0414 (16)	0.0286 (13)	0.0031 (12)	0.0080 (11)	0.0068 (12)
C25	0.0295 (13)	0.0248 (13)	0.0206 (11)	0.0047 (11)	0.0051 (10)	0.0056 (10)
C26	0.0343 (14)	0.0351 (15)	0.0258 (13)	0.0088 (12)	0.0108 (11)	0.0052 (11)
C31	0.0200 (12)	0.0276 (13)	0.0256 (12)	0.0012 (10)	0.0077 (10)	0.0016 (10)
C32	0.0324 (14)	0.0326 (15)	0.0285 (13)	0.0049 (12)	−0.0017 (11)	0.0014 (11)
C33	0.0356 (15)	0.0374 (16)	0.0274 (13)	−0.0011 (12)	−0.0014 (12)	−0.0038 (12)
C34	0.0370 (15)	0.0298 (14)	0.0301 (13)	−0.0030 (12)	0.0057 (12)	−0.0075 (11)
C35	0.0246 (13)	0.0276 (13)	0.0298 (13)	−0.0013 (11)	0.0102 (11)	0.0004 (11)
C36	0.0343 (14)	0.0248 (13)	0.0397 (15)	0.0014 (11)	0.0131 (12)	−0.0024 (12)
C41	0.0199 (12)	0.0292 (13)	0.0277 (13)	−0.0010 (10)	0.0080 (10)	0.0030 (11)
C42	0.0300 (14)	0.0417 (16)	0.0296 (14)	−0.0020 (12)	0.0013 (11)	−0.0009 (12)
C43	0.0268 (14)	0.0500 (18)	0.0313 (14)	0.0018 (13)	−0.0032 (12)	0.0086 (13)
C44	0.0264 (14)	0.0384 (16)	0.0389 (15)	0.0052 (12)	0.0046 (12)	0.0114 (13)
C45	0.0213 (12)	0.0323 (14)	0.0327 (13)	0.0039 (11)	0.0097 (11)	0.0064 (11)
C46	0.0306 (14)	0.0277 (14)	0.0458 (16)	0.0088 (12)	0.0132 (13)	0.0065 (13)
C1	0.055 (2)	0.0455 (19)	0.0450 (17)	−0.0154 (16)	0.0094 (15)	−0.0067 (16)
C2	0.0484 (18)	0.0380 (17)	0.0226 (13)	−0.0190 (15)	−0.0033 (13)	0.0032 (12)
C3	0.0483 (18)	0.0447 (17)	0.0283 (14)	−0.0067 (15)	0.0072 (13)	−0.0054 (13)
C4	0.0443 (17)	0.0364 (16)	0.0299 (14)	−0.0098 (13)	0.0015 (13)	−0.0003 (12)
N1	0.086 (2)	0.062 (2)	0.085 (2)	−0.0259 (18)	0.025 (2)	−0.0363 (19)
N2	0.0455 (16)	0.0587 (17)	0.0381 (14)	−0.0186 (14)	0.0064 (12)	−0.0048 (13)
N3	0.0510 (17)	0.075 (2)	0.0492 (16)	−0.0219 (15)	0.0059 (14)	−0.0160 (15)

### Geometric parameters (Å, º)

**Table:**

Cu1—N10	1.981 (2)	C23—H23	0.9300
Cu1—N20	2.055 (2)	C24—C25	1.409 (3)
Cu1—N30	1.974 (2)	C24—H24	0.9300
Cu1—N40	2.126 (2)	C25—C26	1.431 (3)
Cu1—O1	2.154 (2)	C26—H26	0.9300
Cu1—O2	2.586 (2)	C31—C35	1.398 (3)
O1—N4	1.253 (3)	C31—C41	1.438 (3)
O2—N4	1.238 (3)	C32—C33	1.393 (4)
O3—N4	1.234 (3)	C32—H32	0.9300
N10—C11	1.354 (3)	C33—C34	1.359 (4)
N10—C12	1.334 (3)	C33—H33	0.9300
N20—C21	1.362 (3)	C34—C35	1.399 (3)
N20—C22	1.326 (3)	C34—H34	0.9300
N30—C31	1.361 (3)	C35—C36	1.429 (3)
N30—C32	1.330 (3)	C36—C46	1.351 (4)
N40—C41	1.359 (3)	C36—H36	0.9300
N40—C42	1.327 (3)	C41—C45	1.401 (3)
C11—C15	1.405 (3)	C42—C43	1.400 (4)
C11—C21	1.430 (3)	C42—H42	0.9300
C12—C13	1.397 (4)	C43—C44	1.355 (4)
C12—H12	0.9300	C43—H43	0.9300
C13—C14	1.364 (4)	C44—C45	1.404 (3)
C13—H13	0.9300	C44—H44	0.9300
C14—C15	1.410 (4)	C45—C46	1.434 (4)
C14—H14	0.9300	C46—H46	0.9300
C15—C16	1.435 (4)	C1—N1	1.144 (4)
C16—C26	1.349 (4)	C1—C4	1.411 (4)
C16—H16	0.9300	C2—N2	1.151 (4)
C21—C25	1.401 (3)	C2—C4	1.404 (4)
C22—C23	1.396 (4)	C3—N3	1.147 (4)
C22—H22	0.9300	C3—C4	1.415 (4)
C23—C24	1.366 (4)		
			
N10—Cu1—N30	177.46 (8)	C23—C22—H22	118.5
N10—Cu1—N40	100.85 (8)	C24—C23—C22	119.4 (2)
N20—Cu1—N10	82.20 (8)	C24—C23—H23	120.3
N20—Cu1—N30	95.54 (8)	C22—C23—H23	120.3
N20—Cu1—N40	121.78 (8)	C23—C24—C25	119.7 (2)
N30—Cu1—N40	81.31 (8)	C23—C24—H24	120.2
N10—Cu1—O1	90.15 (8)	C25—C24—H24	120.2
N20—Cu1—O1	145.22 (8)	C21—C25—C24	117.0 (2)
N30—Cu1—O1	91.07 (8)	C21—C25—C26	118.9 (2)
N40—Cu1—O1	92.97 (8)	C24—C25—C26	124.1 (2)
N10—Cu1—O2	90.41 (8)	C16—C26—C25	121.1 (2)
N20—Cu1—O2	93.23 (7)	C16—C26—H26	119.5
N30—Cu1—O2	88.55 (7)	C25—C26—H26	119.5
N40—Cu1—O2	144.19 (7)	N30—C31—C35	122.3 (2)
O1—Cu1—O2	52.76 (6)	N30—C31—C41	117.1 (2)
C12—N10—C11	118.6 (2)	C35—C31—C41	120.6 (2)
C12—N10—Cu1	128.10 (18)	N30—C32—C33	122.4 (2)
C11—N10—Cu1	113.29 (15)	N30—C32—H32	118.8
C22—N20—C21	117.7 (2)	C33—C32—H32	118.8
C22—N20—Cu1	131.50 (18)	C34—C33—C32	119.2 (2)
C21—N20—Cu1	110.75 (16)	C34—C33—H33	120.4
C32—N30—C31	118.5 (2)	C32—C33—H33	120.4
C32—N30—Cu1	126.82 (17)	C33—C34—C35	120.2 (2)
C31—N30—Cu1	114.70 (15)	C33—C34—H34	119.9
C42—N40—C41	117.4 (2)	C35—C34—H34	119.9
C42—N40—Cu1	132.69 (18)	C31—C35—C34	117.3 (2)
C41—N40—Cu1	109.91 (15)	C31—C35—C36	118.8 (2)
N4—O1—Cu1	104.48 (15)	C34—C35—C36	123.9 (2)
N4—O2—Cu1	84.07 (15)	C46—C36—C35	121.1 (2)
O3—N4—O2	121.4 (2)	C46—C36—H36	119.5
O3—N4—O1	120.0 (2)	C35—C36—H36	119.5
O2—N4—O1	118.6 (2)	N40—C41—C45	123.8 (2)
N10—C11—C15	123.1 (2)	N40—C41—C31	117.0 (2)
N10—C11—C21	116.9 (2)	C45—C41—C31	119.2 (2)
C15—C11—C21	120.0 (2)	N40—C42—C43	122.5 (3)
N10—C12—C13	122.1 (3)	N40—C42—H42	118.8
N10—C12—H12	119.0	C43—C42—H42	118.8
C13—C12—H12	119.0	C44—C43—C42	119.9 (2)
C14—C13—C12	119.6 (2)	C44—C43—H43	120.0
C14—C13—H13	120.2	C42—C43—H43	120.0
C12—C13—H13	120.2	C43—C44—C45	119.7 (2)
C13—C14—C15	120.1 (2)	C43—C44—H44	120.1
C13—C14—H14	120.0	C45—C44—H44	120.1
C15—C14—H14	120.0	C41—C45—C44	116.6 (2)
C11—C15—C14	116.6 (2)	C41—C45—C46	119.1 (2)
C11—C15—C16	118.5 (2)	C44—C45—C46	124.2 (2)
C14—C15—C16	124.9 (2)	C36—C46—C45	121.2 (2)
C26—C16—C15	121.4 (2)	C36—C46—H46	119.4
C26—C16—H16	119.3	C45—C46—H46	119.4
C15—C16—H16	119.3	N1—C1—C4	178.9 (4)
N20—C21—C25	123.2 (2)	N2—C2—C4	178.7 (3)
N20—C21—C11	116.7 (2)	N3—C3—C4	178.8 (4)
C25—C21—C11	120.1 (2)	C1—C4—C2	120.6 (3)
N20—C22—C23	123.0 (2)	C2—C4—C3	118.7 (3)
N20—C22—H22	118.5	C3—C4—C1	120.8 (3)

### Hydrogen-bond geometry (Å, º)

**Table:**

*D*—H···*A*	*D*—H	H···*A*	*D*···*A*	*D*—H···*A*
C12—H12···O3^i^	0.93	2.52	3.206 (3)	131
C24—H24···O2^ii^	0.93	2.44	3.231 (3)	144
C36—H36···O3^iii^	0.93	2.45	3.307 (3)	153
C46—H46···O1^iv^	0.93	2.47	3.201 (3)	136

Symmetry codes: (i) −*x*, −*y*, −*z*+1; (ii) −*x*+1, −*y*, −*z*+1; (iii) *x*, *y*+1, *z*; (iv) −*x*, −*y*+1, −*z*+1.

## References

[bb1] Brandenburg, K. (2001). *DIAMOND* Crystal Impact, Bonn, Germany.

[bb2] Chen, Z.-M., Li, W., Yang, Y.-Q., Kuang, D.-Z., Feng, Y.-L., Wang, J.-Q. & Zhang, F.-X. (2005). *Hunan Shifan Daxue Ziran Kexue Xuebao*, **28**, 54–58.

[bb3] Clark, R. C. & Reid, J. S. (1995). *Acta Cryst.* A**51**, 887–897.

[bb4] Golub, A. M., Kőhler, H. & Skopensko, V. V. (1986). In *Chemistry of Pseudohalides* Amsterdam: Elsevier.

[bb5] Janiak, C. (2000). *J. Chem. Soc. Dalton Trans.* pp. 3885–3896.

[bb6] Lacková, K. (2012). Unpublished results.

[bb7] Marjani, K., Davies, S. C., Durrant, M. C., Hughes, D. L., Khodamorad, N. & Samodi, A. (2005). *Acta Cryst.* E**61**, m11–m14.

[bb8] Marsh, R. E. (1997). *Acta Cryst.* B**53**, 317–322.

[bb9] Meerssche, M. van, Germain, G., Declercq, J. P. & Wilputte-Steinert, L. (1981). *Cryst. Struct. Commun.* **10**, 47–53.

[bb10] Ovens, J. S., Geisheimer, A. R., Bokov, A. A., Ye, Z.-G. & Leznoff, D. B. (2010). *Inorg. Chem.* **49**, 9609–9616. 10.1021/ic101357y 20860369

[bb11] Oxford Diffraction (2007). *CrysAlis CCD* and *CrysAlis RED* Oxford Diffraction Ltd, Abingdon, England.

[bb12] Potočňák, I., Burčák, M., Baran, P. & Jäger, L. (2005). *Transition Met. Chem.* **30**, 889–896.

[bb13] Potočňák, I., Vavra, M., Jäger, L., Baran, P. & Wagner, C. (2008). *Transition Met. Chem.* **33**, 1–8.

[bb14] Prasad, B. L. V., Sato, H., Enoki, T., Cohen, S. & Radhakrishnan, T. P. (1999). *J. Chem. Soc. Dalton Trans.* pp. 25–29.

[bb15] Sheldrick, G. M. (2008). *Acta Cryst.* A**64**, 112–122. 10.1107/S0108767307043930 18156677

